# Tau Repeats Promote Amyloid‐β Fibril Disassembly *in Vitro*

**DOI:** 10.1002/alz70861_108195

**Published:** 2025-12-23

**Authors:** Soljee Yoon, Wonbin Seo, InWook Park, Suhyun Ye, Illhwan Cho, Hye Yun Kim, YoungSoo Kim

**Affiliations:** ^1^ Yonsei University, Yeonsu‐gu, Incheon Korea, Republic of (South); ^2^ Yonsei Institute of Pharmaceutical Sciences, Incheon, Incheon Korea, Republic of (South); ^3^ Yonsei University, Incheon, Incheon Korea, Republic of (South); ^4^ Yonsei Institute of Pharmaceutical Sciences, Incheon Korea, Republic of (South); ^5^ Amyloid Solution Inc., Seongnam, Gyeonggi‐do Korea, Republic of (South); ^6^ College of Pharmacy, Yonsei University, Incheon, Incheon Korea, Republic of (South)

## Abstract

**Background:**

Alzheimer’s disease (AD) is a neurodegenerative disorder marked by the pathological accumulation of two key proteins in the brain: amyloid‐β (Aβ) and tau. The Aβ‐tau interaction is known to affect AD through mechanisms that are not fully understood. Consequently, the present study investigated the impact of tau repeats on Aβ aggregation dynamics. Given the inherent structural complexity of tau, we developed a simplified model system that focused on tau repeats.

**Method:**

Direct interaction between Aβ and tau was observed via microscale thermophoresis (MST) and peptide mapping. To further investigate the role of tau in modulating Aβ aggregation, we utilized Thioflavin T (ThT) assays, Sodium Dodecyl Sulfate‐Polyacrylamide Gel Electrophoresis (SDS‐PAGE) / Photo‐Induced Cross‐Linking of Unmodified Protein (PICUP), and Transmission electron microscopy (TEM).

**Result:**

Tau repeats demonstrate binding affinity for Aβ, particularly interacting with hydrophobic domains within Aβ. When further probed, all tau repeats significantly inhibited the fibrillization of Aβ. Notably, tau repeats have been observed to not only dissociate Aβ fibrils but also stabilize oligomer species. Tau repeats with amyloid motifs demonstrated most pronounced effect throughout the experiments.

**Conclusion:**

Collectively, we have demonstrated that tau repeats modulate Aβ aggregation kinetics. These findings advanced the understanding of tau‐Aβ interactions in neurodegeneration and established the foundation for future research tools.

